# Association between Anemia and Cognitive Impairment among Elderly Patients with Heart Failure

**DOI:** 10.3390/ijerph16162933

**Published:** 2019-08-15

**Authors:** Eun Young Kim, Youn-Jung Son

**Affiliations:** Red Cross College of Nursing, Chung-Aug University, Seoul 06974, Korea

**Keywords:** heart failure, aged, anemia, cognitive dysfunction, cross-sectional studies

## Abstract

Cognitive impairment is more prevalent in heart failure (HF) patients. Anemia can influence cognitive ability and is likely more prevalent in elderly patients with HF. However, there are limited data on the association of anemia with cognitive impairment in elderly HF patients. This study aimed to identify the association between anemia and cognitive impairment in elderly HF patients. This secondary data analysis included 181 patients aged 60 years or older with HF. Patients were categorized into an anemic or non-anemic group based on World Health Organization (WHO) criteria. We assessed the cognitive function using the Modified Mini-Mental State (3MS) at the time of enrollment. The prevalence of anemia and cognitive impairment in older patients with HF was the same at 35.4%. The main finding of the multiple logistic regression indicated that compared to a non-anemic status, anemia increased the risk of cognitive impairment (odds ratio (OR) = 4.268, 95% confidence interval (CI) = 1.898–9.593, *p* < 0.001). Healthcare providers should recognize the value of the significance of early assessment of anemic status and cognitive function following HF. A prospective cohort study should identify the pathway of the association between anemia and incidence of cognitive impairment.

## 1. Introduction

The increasing aging population and prevalence of risk conditions (e.g., hypertension, diabetes) have increased the number of persons at risk of HF worldwide [1]. In Korea, these factors are expected to increase the prevalence of HF (heart failure) until 2040 [2].

In particular, HF disproportionately afflicts older adults, and is often more challenging for elderly patients because of multi-morbid illnesses, polypharmacy, and cognitive impairments [3,4]. HF can increase the re-hospitalization rate and mortality risk because of chronic conditions with frequent symptom exacerbations [5,6]. Although medical technology is continuously developing, an improved long-term outcome for HF patients is not yet known [7]. Better HF self-care can reduce mortality hospital admissions [8]. Thus, patient self-care should be emphasized in the HF population [9]. Self-care in HF should involve complex requirements for optimal HF disease management including behavioral adaptation.

Previous studies reported that elderly HF patients might have less optimal self-care because of their cognitive dysfunctions [10]. According to recent studies [11,12], approximately 73–80% of HF patients have cognitive impairment as a consequence of HF, because HF causes chronic or intermittent cerebral hypoperfusion and modification of cerebrovascular reactivity [13,14,15]. Unfortunately, cognitive impairment may remain unrecognized by health professionals caring for older patients with HF, because it falls outside traditional disease-focused perspectives in clinical care [13,15]. Consequently, it is vital to prevent or manage cognitive decline in older adults with HF to improve quality of life and reduce mortality rate.

Anemia prevalence in HF patients ranges from 17–70% [16,17]. Among the elderly, an important public health concern is anemia [17]. Anemia is defined as a hemoglobin level below 13 g/dL for men and below 12 g/dL for women based on the World Health Organization (WHO) criteria [18]. Specifically, in community-based older people, some studies have reported that anemia is related to cognitive decline and dementia [19,20,21,22]; however, few have demonstrated its association with HF [23]. To date, the risk factors of cognitive impairment among elderly HF patients are not well understood, despite being common among this population. Specifically, identifying reversible or modifiable factors of cognitive impairment should be a research priority. To achieve this goal, our study was conducted on the elderly HF population. 

This study aims to investigate the association of anemia with increased risk of cognitive impairment in elderly HF patients.

## 2. Methods

### 2.1. Study Design and Participants

A secondary data analysis was conducted of data from the original study on HF patients’ self-care behaviors. Detailed information on the original study is available elsewhere [24].This cross-sectional study employed a convenience sample from the cardiology outpatient clinic of a tertiary university hospital. The inclusion criteria were that patients had to be aged 60 years or older; diagnosed with HF using echocardiographic findings, clinical history, the presence of symptoms, and diagnoses for at least one year.

Exclusion criteria were a history of a physician-confirmed diagnosis of dementia or mild cognitive impairment presence of paralysis or physical impairment due to a history of stroke; major depressive disorder; terminal illness with a predicted life expectancy of six months or less; and requiring hospice or palliative care.

### 2.2. Measurements

#### 2.2.1. Socio-Demographic and Clinical Characteristics

Information on patients’ socio-demographic characteristics including their age, gender, education, living with a partner, and monthly income was included using a self-reported questionnaire. Clinical characteristics including period after HF diagnosis, the New York Heart Association (NYHA) class, diagnosed co-morbidities, left ventricular ejection fraction (LVEF), level of hemoglobin (Hb) and hematocrit (Hct), and prescribed medication were obtained through an analysis of electronic medical records.

#### 2.2.2. Anemia

Patients were categorized into the anemic or non-anemic group based on the WHO criteria (Hb concentration of less than 13 g/dL for men and less than 12 g/dL for women) [18]. Hb concentration was assessed through medical record abstraction at the time of enrollment in the study.

#### 2.2.3. Cognitive Impairment

Cognitive impairment at the time of enrollment was measured using the Korean version of the Modified Mini-Mental Status Examination (3MS) [25]. The 3MS was developed by Ten and Chui [26] and is a brief and well-established cognitive test compared to the Mini Mental State Exam (MMSE) [25]. The 3MS was useful in and sensitive to the measurement of cognitive function in HF patients [26]. The score of 3MS ranges from 0 to 100 points, and the higher the score, the better is cognitive function [27]. A 3MS score < 80 was considered indicative of cognitive impairment [28,29].

### 2.3. Ethical Considerations and Data Collection

The Institutional Review Board (IRB approval number: 17299) approval was given. Before data collection, participants completed the informed consent form, which included the aim of the study and a guarantee of the anonymity and confidentiality of their information. After completing the informed consent form, participants completed the structured questionnaire.

### 2.4. Data Analysis

Statistical analysis was performed using SPSS/WIN 23.0 software (IBM Corp., Armonk, NY, USA). Continuous variables were analyzed using the mean and standard deviation, and categorical variables by using percentages. A Chi-square test was performed to describe the prevalence of anemia based on patients’ characteristics. The prevalence of cognitive impairment based on patients’ anemia status was calculated through an independent *t*-test and chi-square test. 

To determine the impact of anemia on the cognitive impairment of older adults with HF, a multiple logistic regression analysis was used after an adjusting confounding factors. The odds ratio (OR) and a 95% confidence interval (CI) were observed. A *p* value of less than 0.05 was considered statistically significant.

## 3. Results

### 3.1. Prevalence of Anemia According to Patients’ Characteristics

Regarding patients’ characteristics, the mean age of the 181 participants was 70.01 (± 7.62) years. Furthermore, 75.1% (n = 136) of the participants were men and 54.7% (n = 99) had obtained more than middle school education. Approximately 89.5% of patients were NYHA functional class I and II. For the majority of participants, a period of more than five years had passed after their HF diagnosis (89.5%, n = 162) and having coronary artery disease (CAD) as a comorbidity (75.1%, n = 136). Finally, 34.8% (n = 63) of patients had less than 40% of LVEF. The prevalence of anemia among older patients with HF was about 35.4% (n = 64).

Table 1 presents the prevalence of anemia according to patients’ characteristics. The prevalence of anemia differed significantly according to age, gender, education level, and monthly income. For example, a higher prevalence of anemia was evident among those aged more than 70 years. 

Older female patients also had a higher prevalence of anemia (51.1%, n = 23, *p* = 0.011) than men (30.1%, n = 41). Patients who graduated from elementary school and those who were uneducated were more likely to have anemia (46.3%, n = 38, *p* = 0.016) than those who graduated from middle school or higher (26.3%, n = 26). In addition, anemia was more prevalent among participants with monthly incomes of less than 1,000,000 KRW (42.1%, n = 48, *p* = 0.013).

### 3.2. Prevalence of Cognitive Impairment According to Patients’ Anemic Status

In the study, the prevalence of cognitive impairment of older patients with HF was about 35.4% (n = 64). As shown in Table 2, the average score of cognitive function in HF patients with anemia was lower (78.64 ± 11.61) than that of those in the non-anemia group (86.13 ± 7.68). Regarding prevalence, a higher proportion of patients with anemia (60.9%) have cognitive impairment based on the cut-off of < 80 on 3MS than those in the non-anemia group (24.8%) (χ^2^ = 4.635, *p* < 0.001). 

### 3.3. Influence of Anemia on Cognitive Impairment among Older Adults with HF

The univariate logistic regression models indicated that age, gender, education, monthly income, and anemia were significantly associated with a higher risk of cognitive impairment (*p* < 0.05 for all).

A multiple logistic regression was conducted to determine the extent of this association with anemia. After adjusting for confounding factors, anemia significantly increased the risk of cognitive impairment compared to a non-anemic status (OR = 4.268, 95% CI = 1.898–9.593, *p* < 0.001) (Table 3). Furthermore, our findings revealed that the risk of cognitive impairment was 3.2 times higher among those aged more than 80 years than for those younger than 79 years (OR = 3.208, 95% CI = 1.048–9.818, *p* = 0.041). Finally, patients who graduated from elementary school and those who were uneducated were more likely to have a cognitive impairment (OR = 4.918, 95% CI = 2.195–11.020, *p* < 0.001).

## 4. Discussion

The physical condition of elderly HF patients differs from those of younger patients, entailing a significantly worse prognosis including that of cognitive decline [4]. Recently, the significance of anemia as a risk factor of cognitive impairment in HF patients has been identified [30]. However, the cause of cognitive impairment in terms of anemia is debated [31].

Our main results showed that anemia was significantly linked to cognitive impairment based on a score of 3MS < 80 for older adults with HF. In our study, compared to those in the non-anemia group, the risk of cognitive impairment increased 4.268 times for those with anemia, and remained significant after adjusting for other potential risk factors of cognitive impairment. This finding supports a recent review [32] that confirmed the significant association between anemia and cognitive performance in people aged 65 years and more. Importantly, the adjusted ORs of cognitive impairment were three to four times higher than those in previous studies on chronic HF patients aged 18 years or older [33] and the community-based elderly population [22]. Anemia might directly aggravate the effects of hypoperfusion on cerebral metabolism, decreasing cognitive function [34]. Thus, assessing the presence of anemia may be helpful in evaluating the risk of cognitive impairment of HF patients. Moreover, our findings imply that older adults with HF may be more vulnerable in terms of cardiovascular and non-cardiovascular morbidity and mortality, dependency, and complications relative to the community-dwelling elderly [3]. Elderly HF patients with cognitive impairments have poorer self-care behaviors including adherence to medication adherence, and lifestyle modification, which can lead to an increased risk of hospital admission and mortality [10]. Thus, health professionals should consider assessing both anemic status and cognitive function when designing HF disease management strategies.

In contrast, our results were not consistent with those of similar studies by Payne et al. [35] and Chen et al. [36] on a healthy elderly population. These inconsistent findings may be due to variations in prevalence of anemia between healthy older adults and elderly HF patients. Furthermore, the prevalence of anemia and types thereof might differ according to race and geographic area [22]. However, the anemia prevalence of 35% among the elderly HF patients in this study was slightly lower than the 42% found in a prior study on HF patients [37]. Regarding the characteristics of participants in this study, 89.5% were NYHA I/II and 65% had LVEF > 40%. This is in accordance with evidence that anemia may be associated with a lower LVEF value and higher NYHA classification, highlighting a severe functional limitation [38].

HF with preserved EF (HFpEF) is diagnosed using patients’ HF symptoms and signs with normal or near normal LVEF [39]. The proportion of HFpEF is increasing steady, especially among elderly females [40]. In our study, around 65% of participants may be classified as HFpEF based on the LVEF criteria. However, we could not find accurate classification evidence in the medical charts for HFpEF versus HF with reduced EF (HfrEF). To date, there is no universally agreed upon definition of HFpEF [39]. However, according to previous studies [39,40], anemia is more prevalent in HFpEF. Thus, further studies are needed to identify the combined effect of anemia and higher grade of NYHA function on cognitive function among HFpEF and HFrEF.

Unsurprisingly, our multiple logistic regression analysis indicated that older age and a lower level of education were associated with cognitive impairment in HF elderly patients. This finding is aligned with recent reviews confirming age and education level as factors influencing cognitive impairment in HF [13,41]. Being elderly is associated with physiological and general health deterioration including physical functioning, understanding health information, and limited health access [41]. In addition, a low level of education is consistent with a lower level of HF knowledge and awareness, which increase patients’ independence and confidence and consequently affects their cognitive function [42]. Therefore, health professionals need to recognize the consequences of older age and a low level of education as well as anemic conditions to adopt strategies to minimize its negative effect on patients’ health outcomes such as cognitive impairment. 

Regarding cognitive impairment, our study revealed a prevalence of 35.4% among elderly HF patients. Gure et al. [43] reported that a prevalence of mild cognitive impairment and dementia were 24% and 15% relatively among older adults with HF. According to Callegari et al. [44], the prevalence of cognitive impairment was about 26% of HF participants in one cognitive function. Although the 3MS screening tool is more advantageous than the MMSE for an HF population [45,46], the 3MS can be also influenced by age, gender, education level, and comorbidities. Accordingly, a more precise instrument may enable a better diagnosis of cognitive impairments among HF patients. Furthermore, researchers should design special tools for evaluating the cognitive function of patients with HF. In particular, evaluating all risk factors for cognitive decline after HF can help prevent cognitive impairment among older adults with HF. 

Our study has some limitations. First, this study used convenience sampling, which can cause sampling bias. In addition, the majority of participants were male and an NYHA functional class I and II, meaning relatively low severity. The mean age of participants was relatively young, and the average period of the study was shorter than that of previous research, which can influence the severity of HF [47]. This can hinder recruiting participants closer to the population. Second, the prevalence of anemia and cognitive impairment may differ among elderly patients with HFpEF and HFrEF. Thus, future studies should consider the type of HF. Third, this study was conducted at a single center in Korea. This may limit the generalizability of findings to various situation such as other countries and different healthcare settings. Fourth, we used a single screening tool using the 3MS to evaluate cognitive impairment after HF. Although the 3MS is a valid and proprietary instrument for detecting the presence of cognitive impairment, other screening tests or multiple cognitive assessment tools are comparably effective for a more in-depth assessment.

## 5. Conclusions

Our study confirmed the significant association of anemia with cognitive impairment following HF in elderly patients. As a modifiable risk factor, considering the screening of anemia when diagnosing HF can play a role in evaluating the risk of cognitive impairment HF among elderly patients. Moreover, both anemia and cognitive impairment in HF may influence the ability to perform HF self-care activities. Thus, health professionals should be aware of the importance of a periodic assessment for anemic and cognitive function status among elderly HF patients based on their age and level of education. Longitudinal studies are needed to increase our knowledge on the impact of anemia on adverse health outcomes including cognitive impairment among elderly patients with multi-comorbidities such as stroke and major depressive disorders.

## Figures and Tables

**Table 1:** Prevalence of anemia according to patients’ characteristics (n = 181).

Characteristics	Total (n = 181)	Anemia (n = 64)	Non-anemia (n = 117)	χ^2^	*p* Value
	n (%) or Mean *± SD*	n (%)	n (%)		
Age (yrs)	70.01 ± 7.62				
60–69	100 (55.2)	19 (19.0)	91 (91.0)	27.002	< 0.001
70–79	52 (28.7)	27 (51.9)	25 (48.1)		
≥ 80	29 (16.0)	18 (62.1)	11 (37.9)		
Gender					
Men	136 (75.1)	41 (30.1)	95 (69.9)	6.052	0.011
Women	45 (24.9)	23 (51.1)	22 (48.9)		
Education					
≤ Elementary school	82 (45.3)	38 (46.3)	44 (53.7)	8.315	0.016
≥ Middle School	99 (54.7)	26 (26.3)	73 (73.7)		
Living with partner, yes	143 (79.0)	46 (32.2)	97 (67.8)	3.035	0.081
Monthly Income (10,000 KRW)					
< 100	114 (63.0)	48 (42.1)	66 (57.9)	6.132	0.013
≥ 100	67 (37.0)	16 (23.9)	51 (76.1)		
NYHA class					
I	56 (30.9)	18 (32.1)	38 (67.9)	1.462	0.481
II	106 (58.6)	37 (34.9)	69 (65.1)		
III–IV	19 (10.5)	9 (47.4)	10 (52.6)		
Period after HF diagnosis (yrs)	5.02 ± 14.84				
< 5	19 (10.5)	11 (57.9)	8 (42.1)	3.68	0.055
≥ 5	162 (89.5)	53 (32.7)	109 (67.3)		
HTN, yes	88 (4.6)	37 (42.0)	51 (58.0)	3.35	0.067
DM, yes	55 (30.4)	20 (36.4)	35 (63.6)	0.035	0.952
CAD, yes	136 (75.1)	46 (33.8)	90 (66.2)	0.564	0.453
LVEF (%)	42.81 ± 9.33				
≤ 40	63 (34.8)	25 (39.7)	38 (60.3)	0.79	0.374
> 40	118 (65.2)	39 (33.1)	79 (66.9)		
ACEI/ARB, yes	67 (37.0)	21 (31.3)	46 (68.7)	1.189	0.276
Beta blocker, yes	117 (64.6)	23 (35.9)	41 (64.1)	0.014	0.904
Lasix, yes	64 (35.4)	36 (30.8)	81 (69.2)	3.05	0.081

NYHA: New York Heart Association; HTN: hypertension; DM: diabetes mellitus; CAD: coronary artery disease; LVEF: left ventricular ejection fraction; ACEI: angiotensin converting enzyme inhibitor; ARB: angiotensin receptor blocker.

**Table 2:** Prevalence of cognitive impairment according to patients’ anemic status (n = 181).

Cognitive Function	Total (n = 181)	Anemia (n = 64)	Non-anemia (n = 117)	χ^2^ or t	*p* Value
	n (%) or Mean ± SD	n (%) or Mean ± SD	n (%) or Mean ± SD		
3MS score	84.49 ± 9.90	78.64 ± 11.61	86.13±7.68	23.052	<0.001
< 80 (Impairment)	64 (35.4)	39 (60.9)	29 (24.8)	4.635	<0.0001
≥ 80 (Normal)	117 (64.6)	25 (39.1)	88 (75.2)		

3MS: The Modified Mini-Mental State

**Table 3:** Multiple logistic regression analysis of cognitive impairment among older adults with heart failure (*n* = 181).

Variables	Categories	Unadjusted Model		Adjusted Model	
		OR (95% CI)	*p* Value	OR (95% CI)	*p* Value
Age (yrs)	≤ 79	1	< 0.001	1	0.041
	≥ 80	9.115 (3.477–23.891)		3.208 (1.048–9.818)	
Gender	Men	1	0.005	1	0.684
	Women	2.703 (1.356–5.391)		1.194 (0.508–2.809)	
Education	≥ Middle School	1	< 0.001	1	< 0.001
	≤ Elementary school	7.935 (3.992–15.775)		4.918 (2.195–11.020)	
Monthly Income (KRW)	≥ 1,000,000	1	< 0.001	1	0.239
	< 1,000,000	4.425 (2.145–9.132)		1.703 (0.702–4.130)	
Anemic status	Non-anemia	1	< 0.001	1	< 0.001
	Anemia	4.734 (2.460–9.108)		4.268 (1.898–9.593)	

OR: Odds Ratio; CI: Confidence Interval; 3MS: The Modified Mini-Mental State
